# Identification of Factors Associated with Potential Doping Behavior in Sports: A Cross-Sectional Analysis in High-Level Competitive Swimmers

**DOI:** 10.3390/ijerph15081720

**Published:** 2018-08-10

**Authors:** Sime Devcic, Jakob Bednarik, Dora Maric, Sime Versic, Damir Sekulic, Zvonimir Kutlesa, Antonino Bianco, Jelena Rodek, Silvester Liposek

**Affiliations:** 1 Special Hospital Biograd n/m, 23120 Biograd, Croatia; cicved@gmail.com; 2 Faculty of Medicine, University of Mostar, 88000 Mostar, Bosnia and Herzegovina; 3 Faculty of Sport, University of Ljubljana, 1000 Ljubljana, Slovenia; jakob.bednarik@fsp.uni-lj.si; 4 PhD Program in Health Promotion and Cognitive Sciences, Sport and Exercise Research Unit, Department of Psychological, Pedagogical and Education Sciences, University of Palermo, 90144 Palermo, Italy; dora.maric@unipa.it (D.M.); antonino.bianco@unipa.it (A.B.); 5 Faculty of Kinesiology, University of Split, 21000 Split, Croatia; sime.versic@gmail.com (S.V.); jrodek@kifst.hr (J.R.); 6 Clinical Hospital Split, 21000 Split, Croatia; zvonimir.kutlesa@gmail.com; 7 Faculty of Mechanical Engineering, University of Maribor, 2000 Maribor, Slovenia; silvester.liposek@um.si; 8 Slovenian Swimming Federation, 1000 Ljubljana, Slovenia

**Keywords:** performance enhancing drugs, predictors, doping behavior, sport achievement, factors of hesitation

## Abstract

*Background*: Doping behavior, including the misuse of performance-enhancing drugs, is currently a serious problem in sports, and the efficacy of preventive efforts directly depends on information regarding the associations among different precipitating factors (PF) and doping behavior. This study aimed to establish the PF of potential doping behavior (PDB) in competitive swimmers. *Methods*: The study included 301 swimmers from Slovenia (153 females, 16.4 ± 2.4 years), tested during the 2017 National Championship. Variables were collected by previously validated questionnaires, which included questions on sociodemographics, sports-related factors, consumption of dietary supplements, knowledge of doping, factors of hesitation, and doping-related factors (i.e., number of doping tests, opinion about the presence of doping in sport). The PDB (positive, neutral, or negative intention toward doping) was observed as a criterion, while other variables were included as predictors in multinomial regression analyses (with “negative” as reference value), which additionally controlled for gender and age category (cadet-, junior-, and senior-level) as confounders. *Results*: The results confirmed higher susceptibility to doping in males (positive: odds ratio (OR): 2.77, 95% confidence interval (CI): 1.27–6.04), those swimmers who reported higher alcohol consumption (neutral: OR: 2.18, 95%CI: 1.06–4.16, positive: OR: 2.14, 95%CI: 1.05–4.37), and those regularly used dietary supplements (positive: OR: 3.62, 95%CI: 1.25–10.52). Competitive achievement in Olympic- (neutral: OR: 0.57, 95%CI: 0.41–0.81, positive: OR: 0.59, 95%CI: 0.39–0.88), and non-Olympic disciplines (positive: OR: 0.54, 95%CI: 0.35–0.83) was protective against PDB. Swimmers who were more concerned about the negative social consequences of doping behavior (i.e., condemnation by family and friends) were less likely to declare a positive intention toward the PDB (family condemnation: OR: 0.69, 95%CI: 0.56–0.86, friends’ condemnation: OR: 0.65, 95%CI: 0.52–0.80). *Conclusions*: The results of the study provide more precise insight into the specific factors associated with PDB in swimming. The established precipitating factors should be incorporated into targeted anti-doping campaigns in this sport.

## 1. Introduction

Doping-behaviors in sports can be generally defined as the violation anti-doping rules, which include: (1) consumption of prohibited performance-enhancing substances (e.g., drugs) and/or (2) the application of other prohibited techniques (e.g., the use of agents that prevent regular procedures from tracing banned substances in athletes’ specimens) [1,2]. Although doping, by its definitions, includes different methods, the majority of doping behavior in sports is related to the misuse of performance-enhancing drugs [2]. Doping behavior is a serious health-threatening behavior with numerous negative consequences for athletes’ health status, including acne, virilization, infertility, subdural hematomas, tendon injuries, altered liver and kidney function, peripheral edema, cardiac hypertrophy, myocardial ischemia, thrombosis, and cardiovascular disease, among others [3,4,5,6]. Ultimately, the consumption of doping drugs is connected to several deaths in athletes [7]. Consequently, the global fight against doping is one of the most important issues in the sports world today. Generally, two approaches in global efforts against doping are recognizable, the first of which could be observed as more “punitive” and the second as more “preventive”. 

From the 1970s onward, most national and international sporting bodies passed rules under which their members agreed to submit to drug testing and to the imposition of sanctions [8]. The World Anti-Doping Agency (WADA) is a body established by International Olympic Committee in order to find the most efficient ways in global fight against doping in sports. Consequently, the WADA established a rigorous detection-based system which consists of testing in order to detect the presence of prohibited substances in athletes’ specimens (i.e., urine or blood). The penalties for doping offenders regularly include banning from competition and stripping of the records and medals obtained [5]. 

Another approach in the global fight against doping is more preventive and includes the following: (1) the identification of the factors associated with doping (either to potential or actual doping behavior), which allows for (2) the identification of specific athletes at risk of doping, and (3) the development of the specific and targeted campaigns aimed at the prevention of doping, which is basically similar to prevention strategies against substance misuse in the public health sector [9,10,11]. In sports, numerous factors have been investigated as being potentially predictive of (i.e., factors of increased risk) or protective against doping behavior in athletes [12,13]. Among these factors, the specific associations were studied for different sociodemographic [13,14], sports-related [13], sociopsychological [15], motivational [16], and coaching and training-related factors [1,17,18]. 

Briefly, the potential doping behavior (PDB) of sailing athletes was non-significantly associated to sport factors, knowledge on nutrition and doping, consumption of the dietary supplements, and various doping-related factors (number of doping testing, opinion on doping presence in sailing). However, the PDB of sailing-athletes was very low (less than 2% declared positive PDB), which almost certainly contributed to non-significant association between studied factors and PDB in this sample [19]. Further, there is no consistency in reports about gender as a factor of influence on doping susceptibility. Notably, while some reports suggest that males are more prone to doping [20,21], higher doping susceptibility in males is not always supported, while some reports evidenced even higher risk for doping vulnerability in females than in males [22]. Meanwhile, studies are consistent with regard to association which exists between personal opinion about doping-presence in sport and doping susceptibility, with higher likelihood of doping behavior in athletes who are convinced that doping is present in their sport [20,21,23,24]. Rugby players were more prone to doping if they were non-smokers, less experienced, and less successful in rugby [23]. A study on international tennis players identified lower doping vulnerability for players who had better knowledge on doping/sport nutrition and who achieved better competitive results [25]. Conversely, sport-achievement was inversely related to doping vulnerability in table-tennis players with higher likelihood of doping behavior in professional-players than in semi-pros and amateurs [14]. In the studies done on college-level athletes, authors evaluated different negative consequences of doping behavior (i.e., health-related problems associated with doping, negative social consequences, sports-related consequences) and confirmed that negative social consequences, including condemnation by religious organizations (i.e., the church) and bad image in the media, are highly prioritized as being a factors of hesitation against doping-behavior [8,26]. 

Studies frequently investigated factors associated to doping susceptibility in sport, but most of these investigations observed athletes involved in different sports as a sample of subjects [27,28,29,30,31,32]. Although findings of these studies are undoubtedly important, such investigations lack some important information. Namely, it is well known that some types of sports reflect specific sociodemographic profiles of participants [33,34]. Athletes involved in different sports differ in their motivational factors [35], while sport-factors (i.e., competitive results, organizational structure) are dissimilar among sports [5,13,19]. Therefore, if investigation observe athletes from different sports and sport-disciplines as unique samples of participants, it is almost certain that previously mentioned differences among sports would directly and/or indirectly have a certain confounding effect on established relationships between studied factors and doping susceptibility. Importantly, such a confounding effect is hardly detectable, because it is mainly a consequence of (1) number of athletes from each sport included in observed sample, and (2) between-sport differences in observed characteristics. Finally, doping susceptibility is known to be different among sports which is clearly supported in regular WADA reports on positive analytical findings, but also in studies which compared doping susceptibility among sports [13,20,22,36]. Consequently, a sport-specific approach is becoming prevalent in studying factors associated to doping susceptibility [1,21,22,37,38,39]. 

Swimming is one of the most popular sports in the world today and is considered one of three “elementary sports” (together with athletics and gymnastics). At the same time, because of its high level of popularity and the importance of highly developed physical capacities to the sport, doping behavior in swimming is relatively frequent [36]. Therefore, those involved in the sport of swimming in general are constantly trying to find the most effective preventive campaign against doping [40]. Meanwhile, to the best of our knowledge, only two studies have specifically investigated the problem of doping in swimming [1,2]. Briefly, Sajber et al. tested the knowledge of swimming coaches and their athletes about doping and identified the necessity of permanent education on the problem for athletes and (older) coaches [2]. In a very recent study, Liposek et al. observed senior swimmers (+18 years of age) and the investigated factors of coaching strategy and training methodology as correlates of potential doping behavior [1]. It is important to note that both of these studies noted an alarming trend in athletes’ opinions about the presence of doping in swimming (i.e., more than one-third of tested athletes were of the opinion that doping is frequent in swimming) [1,2]. 

From previous literature overview, it is evident that associations between investigated precipitating factors (PF) and doping vary with respect to type of sport, sociocultural environment, gender, and competitive level of athletes [13,41]. Therefore, studies that intend to investigate the associations that may exist between different PF and doping behavior in sports should, at minimum, have the following characteristics: (1) be culturally specific (with regard to nation, or at least be specific to the sociocultural environment); (2) be sport-specific; and (3) take into account the possible confounding influence of age and gender on doping behavior in athletes [1,13]. 

This study aimed to evaluate factors associated with potential doping behavior (PDB) in competitive swimmers. Specifically, on the basis of previous studies that examined the problem of factors of influence on PDB, we observed sport-specific variables, sociodemographic indices, knowledge on performance-enhancing drugs and doping, and factors of hesitation against doping as specifically related to PDB in swimmers. The initial hypothesis of the study was that the studied factors will be specifically associated with PDB in swimmers. 

## 2. Materials and Methods 

### 2.1. Participants and Testing

In this study, we tested 301 swimmers from Slovenia (153 females, 16.4 ± 2.4 years old on average). All athletes were tested during the 2017 National Championship. An invitation to participate in the study was sent by the National Swimming Federation to all athletes competing at the championship and was accepted by all; therefore, all swimmers participating were included (please see end of the variables subsection for non-responders rate). The study was originally initiated and approved by the national swimming federation, complied with all ethical guidelines, and received approval from the Institutional Ethics Review Board at the corresponding author’s institution (Ethical Board Approval 10/09/2014-1). All participants older than 18 years of age signed the consent form for their anonymous participation in the study, while one parent/responsible adult signed the consent form for those younger than 18 years of age. 

### 2.2. Variables

Variables were collected by previously validated questionnaires: (1) Questionnaire of Substance Use (QSU); (2) Knowledge of Doping and Performance-Enhancing Drugs (KD); and (3) Questionnaire on Factors of Hesitation (QFH) [1,2,8,20,26]. 

The QSU includes questions on sociodemographic variables; sports-related factors; consumption of dietary supplements, cigarettes and alcohol; and doping factors. The sociodemographic data included the following: age (in years; later additionally categorized into competitive age groups: cadets, juniors, seniors), gender (male or female) and education level (including “elementary school”, “high school”, “student”, and “college-university level”). Sports-related factors were assessed by questions on the following: (1) the athlete’s experience in swimming (in years); (2) age when swimming training was started; and (3) competitive results achieved in (a) non-Olympic events (25-m pool) and (b) Olympic events (50-m pool) (“regional-level medalist”, “national championship—finals”, “national championship—medal”, “European and world championship—participant”, “European and world championship—finals”, “Olympics”). Dietary supplement usage was assessed using the response to one main question (“yes”, “from time to time”, and “no”) and additional questions in which participants were asked about the type of dietary supplements they had used (not reported here). Alcohol consumption was assessed by one query with four possible answers (“I do not drink alcohol”, “I drink alcohol but never binge”, “binge drinking once a month or so”, and “binging more than once a month”). Cigarette smoking was assessed by one question with four possible answers (“I do not smoke”, “I smoke from time to time, but not daily”, “less than 10 cigarettes per day”, and “more than 10 cigarettes per day”). Doping-related factors included different variables related to doping in sport as well as athletes intentions to use doping. Specifically, athletes were asked about the following: (1) the occurrence of doping in swimming (“I do not think doping is used in swimming”, “not sure about it”, “it occurs, but rarely”, and “doping occurs often”); (2) the number of times they have undergone testing for doping (“never tested”, “once or twice”, and “three times and more”); (3) personal PDB (“I would use doping if it would help me”, “not sure” and “I do not intend to use it”); (4) their personal opinion about the main problem of doping in sports (“it is mainly health-threatening behavior”, “it is against fair play”, “equally important”); and (5) their personal opinion about penalties for offenders of anti-doping regulations (“lifelong suspension”, “milder punishment for the first time, then lifelong suspension”, “suspension for a couple of seasons”, “financial punishment”, and “no punishment/should be allowed”). For the purpose of multinomial regression analysis, the responses for PDB were specifically clustered (see text on statistical analyses). In general, the PDB was found to be valid in different sports, including tennis, synchronized swimming, and various team sports [8,13,20,42]. It is also important that recent investigations conducted on team sport athletes of both genders have confirmed a high correlation between PDB and another commonly used measurement tool (the Performance Enhancement Attitude Scale—PEAS) [20,43]. 

The KD has been previously used in various samples of competitive athletes and has been found to be a reliable and valid measuring tool in the evaluation of knowledge of performance-enhancing drugs and doping regulations in different sports, including swimming [2,8,20,26,42]. This measurement-tool consists of 10 questions about problems related to misuse of performance enhancing drugs and anti-doping regulations. Each statement was in a “true (T) or false (F)” form; with one point scored for correct answers. Consequently, the lowest theoretical result was “0”, while the maximal was “10” (i.e., all correct answers). The questions were as follows: (1) Diuretics are considered doping because of their influence on body weight reduction (F); (2) Doping control officers should notify athletes of their testing intentions a few hours prior to any testing (F); (3) If an athlete has an out-of-competition doping test, four weeks should elapse before their next doping test (F); (4) If a doping control officer does not provide valid proof of identity, an athlete can refuse to participate in the testing (T); (5) A “masking agent” is someone who helps an athlete hide their use of doping and is therefore equally responsible for doping offenses (F); (6) The use of doping among team sport athletes, e.g., amphetamines in cycling, has been related to several cases of death due to cardiovascular failure (T); (7) The use of amphetamines by women is related to male-like changes in body appearance (F); (8) Synthetic testosterone (i.e., steroids) increases the quantity of erythrocytes and is therefore common in endurance sports and not prevalent in strength/power sports (F); (9) Use of synthetic testosterone (i.e., steroids) inhibits the production of natural (endogenous) testosterone (T); and (10) When an athlete reports undergoing official medical treatment, he/she cannot be tested for doping (F) [2,8,20,26,42]. The composite score (minimum 1; maximum 10) was calculated and used for analyses in this study. 

The QFH consisted of 19 questions in which participants were asked about their personal opinions on the negative consequences of doping behavior [8,26]. Personal opinions about each of the consequences were assessed on a scale ranging from −3 to +3, where −3 denoted that the participant judged that consequence as absolutely unimportant in their hesitation toward engaging in doping behavior (“It does not upset me at all. I do not care at all”) and +3 denoted that the query had the highest personal importance in relation to that participant’s hesitation toward doping (“This consequence is highly important”). The questions on the consequences of doping behavior included the following: (1) condemnation by family members; (2) condemnation by friends; (3) condemnation by the public (i.e., beyond family or friends); (4) condemnation by religious institutions; (5) the negative image that will be created in media; (6) underestimation of “clean” results, related to the general opinion that even previous results are achieved through doping usage; (7) possible negative financial consequences (direct and/or indirect); (8) eventual imprisonment because of the use of illegal drugs; (9) disqualification from competition; (10) disqualification of previously achieved results; (11) potential problems with future employment (mostly in sports but also in general) because of the negative reputation associated with involvement in doping; (12) behavioral disorders, such as aggressiveness, groundless violence, euphoria, insomnia, depression, and nervousness; (13) psychological addiction to doping drugs because of the performance-enhancing effects; (14) hormonal dysfunctions resulting in a decrease in the size of the testicles, disappearance of sperm in the semen, reversible sterility (in males), inhibition of ovulation (in females), diabetes, and acne; (15) problems with the vital organs, such as anomalies in the function of the liver, benign and malignant liver tumors, excessive blood cholesterol levels, and prostate cancer; (16) cardiovascular problems, including hypertension spells, infarction of the myocardium, cardiac insufficiency, cardiac rhythm problems, and circulatory collapse; (17) body deformities, such as virilization or masculinization in women (appearance of body hair in regions that are normally hairless, such as the face, regions between and around the nipples, back, shoulders, the backs of thighs, and infra-umbilical and intergluteal regions, as well as the development of male pattern baldness), and feminization in males (breast development and high-pitched castrato-like voice), (18) weakening of immune function, with decreased resistance to infections and general immunity problems, and (19) self-disappointment, or feelings of self-failure [8,26]. For the purpose of presentation of results and statistical analysis the originally obtained numerical scores for the QFH (i.e., from “−3” to “+3”; see previous), were transformed in numerical values ranging from 1 (for “−3”) to 7 (for “+3”). The QFH was previously proven to be a reliable and valid measurement tool in studies on competitive athletes, and details are provided elsewhere [8,26].

The questionnaires were provided in Slovenian language, after being translated from Croatian originals by authors of the study. Before testing all athletes were informed that testing was anonymous, and that they can refuse to participate, that they can leave some questions or entire questionnaire unanswered. There was no incentive structure in place, and athletes were informed that there will be no consequences if they will not participate in the study. Athletes were tested in a groups of at least five participants, and after finished they placed questionnaires in closed box. Only two athletes returned questionnaires unanswered. 

### 2.3. Statistics

The parametric/nonparametric nature of the variables was checked using the Kolmogorov–Smirnov test, and statistics included counts and frequencies (Supplementary Tables S1 and S2). Differences between genders and among age-categories (cadets, juniors, seniors) in PDB were established by Chi square test (χ^2^). To establish the relationships between variables and the criterion (PDB), multinomial regression models were employed. The criterion was observed as multinomial response and included three responses: (1) negative PDB (those who responded “I do not intend to engage in doping in the future”); (2) neutral PDB (“not sure”); and (3) positive PDB (“I would engage in doping if it would help me”). The negative PDB was set as the reference value. Additionally, significant correlates of PDB were included in multivariate multinomial regression to establish the unique contribution of each significant predictor. Previous studies have frequently reported significant associations between sociodemographic variables and personal opinions on the presence of doping in sports and PDB [8,20]. Therefore, to obtain possible effect of age (i.e., age-category), and gender (males vs. females) multinomial regressions were adjusted for gender and age-category (e.g., cadet-, junior-, senior-level) as possible confounding factors. For all analyses, Statistica 13.0 (Dell, Tulsa, OK, USA) was used, and *p* < 0.05 was applied. 

## 3. Results

**Figure 1:**
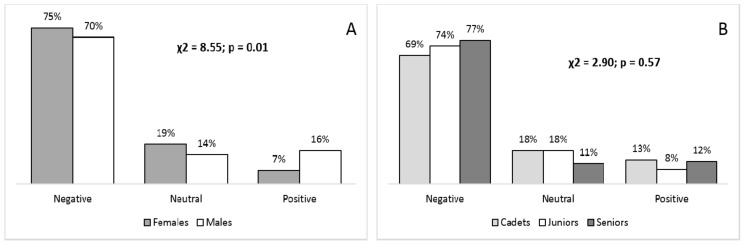
Potential doping behavior (positive, neutral, negative intention) according to gender (**A**); and competitive age-categories (**B**), with Chi squared (χ2) differences.

Males were more prone to PDB than females (χ^2^ = 8.55, *p* = 0.01), while the PDB was not significantly different across age categories (χ^2^ = 2.90, *p* = 0.57) (Figure 1).

The higher odds for positive PDB was found in males (odds ratio (OR): 2.77, 95% confidence interval (CI): 1.27–6.04), while those swimmers who achieved better competitive results were less prone toward PDB. Specifically, the results achieved in Olympic disciplines (e.g., 50-m pools) was correlated to lower likelihood of neutral PDB (OR: 0.57, 95%CI: 0.41–0.81), and positive PDB (OR: 0.59, 95%CI: 0.39–0.88), while competitive result in non-Olympic disciplines (e.g., 25-m pools) was correlated to lower likelihood of positive PDB (OR: 0.54, 95%CI: 0.35–0.83) (Table 1). 

Those swimmers who reported regular usage of the dietary supplements were more likely to be positively oriented toward PDB (OR: 3.62, 95%CI: 1.25–10.52). Neutral PDB was more prevalent in swimmers who were oriented toward less-rigid penalties for doping-offenders (OR: 1.62, 95%CI: 1.09–2.40). Alcohol consumption (binge-drinking) was correlated to PDB, with higher likelihood for neutral PDB (OR: 2.18, 95%CI: 1.08–4.16), and positive PDB (OR: 2.14, 95%CI: 1.05–4.37) in those who reported binge drinking, irrespective of age-category and gender (Table 2). 

Of 19 observed factors of hesitation against doping, those related to negative social- consequences of doping were significantly correlated to PDB. The lower positive PDB was evidenced for swimmers who were concerned about potential condemnation by family members (OR: 0.69, 95%CI: 0.56–0.86), condemnation by friends (OR: 0.65, 95%CI: 0.52–0.80), and condemnation by the public (OR: 0.76, 95%CI: 0.63–0.90) (Table 3). 

Table 4 presents results of multivariate multinomial regression calculation where we included only those variables which were identified as being significantly correlated to PDB in univariate regression models (Table 1, Table 2 and Table 3). The higher odds for neutral PDB was found in those swimmers who were not successful in Olympic disciplines (OR: 0.34, 95%CI: 0.18–0.63), who tended towards less rigid penalties for doping offenders (OR: 1.69, 95%CI: 1.08–2.63), and who binged more often (OR: 1.87, 95%CI: 1.02–3.77). Meanwhile, the unique significant predictors for positive PDB was consumption of dietary supplements, with higher odds for positive PDB in swimmers who regularly consumed dietary supplements (OR: 5.86, 95%CI: 1.67–20.62). 

## 4. Discussion

This study aimed to evaluate the factors related to PDB in competitive swimmers. In general, the main hypothesis is supported, since studied factors were specifically correlated to PDB in this sample of participants. There are several important findings with regard to the study aims, which will be discussed in the forthcoming text. First, lower susceptibility to doping is found in those swimmers who were more successful (i.e., who achieved better competitive results). Second, athletes who reported higher alcohol consumption and more frequent consumption of the dietary supplements are found to be at specific risk for a higher likelihood of PDB. Finally, of all studied factors of hesitation toward doping behavior, only the variables explaining the negative social consequences were evidenced as significantly correlated with PDB in the participating swimmers. In general, swimmers who reported higher concern regarding the negative social consequences of doping were less susceptible to PDB.

### 4.1. Competitive Results and Doping

The competitive results achieved in Olympic- (50-m swimming pools) and non-Olympic disciplines (25-m pools) are found to be protective against PDB, irrespective of age and gender (i.e., covariates did not significantly contribute to the established correlation between competitive results and PDB). Sports achievements are frequently studied in relation to PDB, but the results are not fully consistent [8,14,20]. For example, in a study conducted with rugby players, the authors confirmed a higher likelihood of PDB in those who achieved lower competitive results [8], which is consistent with the findings of studies that investigated sailing athletes [19], synchronized swimmers [13], and male team-sport athletes [20]. Meanwhile, competitive results were not correlated with PDB in male and female kickboxers [41] and female team-sport athletes [20]. Further, in a study performed among table tennis players, the authors showed an increased probability of PDB with a higher sport status [14]. However, it must be noted that in the table tennis study, competitive results were evidenced on a scale showing “sports-related status” (i.e., amateur, semipro, professional), which is only theoretically associated with achievement in competitive sports. Therefore, our results, which indicated a lower likelihood of doping among more successful swimmers, are consistent with those of the majority of studies that have examined this issue, in which a lower susceptibility to doping is found among athletes who achieved better competitive results [8,19,20]. 

Swimming is one of the more objective sports, since competitive results are reliably measured by relay times. Therefore, athletes themselves are able to estimate his/her own athletic capacity, irrespective of factors that may affect athletic achievement in other sports (i.e., the athletic capacity of teammates in team sports, the quality of the slope in skiing, the quality of the opponent in martial arts). In the meantime, it is not unlikely that less capable swimmers will recognize their relative physical and/or competitive “inferiority” as an issue that could be effectively overcome by the use of performance-enhancing drugs (i.e., doping), and this problem has already been reported and discussed in other sports [8]. By all means, such a viewpoint (i.e., acceptance of doping in those who are not satisfied with own achievement) is partially mediated by “real” inferiority and attributed to those athletes who are lacking the physical, mental, and/or other sport-related potential required for advanced achievement [8]. We are all witnessing that competitive sports are easily symbolized by the Spartans’ “Ē tan ē epitas”, whereby those who cannot reach the targeted achievement are left behind. It is easy to imagine the disappointment and even frustration of athletes who are trying hard but are not achieving the results they are striving for. 

As support for previous discussion, it must be stated that the association between certain types of dissatisfaction and positive attitudes toward doping is not characteristic of only the athletic population (i.e., competitive athletes). For example, an Australian study observed male adolescents and showed the relationship between body dissatisfaction and attitudes towards doping in sports. In their findings, the authors, among others, reported that the participants who were more dissatisfied with their bodies were at the same time more likely to support the use of doping in sports [44]. Regardless of the background for the previously discussed findings on the higher susceptibility to doping among swimmers who achieved lower competitive results, it is clear that in the development of accurate and meaningful anti-doping campaigns in swimming, special attention must be paid to swimmers who are not successful, irrespective of their age and gender.

### 4.2. Alcohol Consumption and Doping

Our results indicated higher likelihood of PDB in those athletes who consume alcohol more frequently, and to the best of our knowledge this is the first study to report a significant association between alcohol consumption and doping susceptibility thus far. More precisely, the relationship between “everyday substances”, such as alcohol and cigarettes, and doping susceptibility has not been studied until recently [5,8]. In a study performed on athletes involved in Olympic racket sports, the authors found no correlation between binge drinking and PDB [5], which supports the findings of the study that investigated the same issue in rugby players [8]. Interestingly, in both of these studies, alcohol consumption was very high, with 30–40% of participants binging at least once a month [5,8]. In both mentioned studies, such high alcohol consumption is explained as a way of dealing with the competitive stress and as a regular part of post-sport gatherings [5,8]. Meanwhile, in our study, the consumption of alcohol was much lower than among athletes playing racket sports and rugby (<5% of swimmers reported binge drinking at least once a month), which could partially explain even the correlation between binge drinking and doping susceptibility. The specific explanation is provided in the following text. 

In short, the sport-specific environment and culture often promote alcohol drinking as a behavioral norm [45,46,47]. This finding has been noted even in studies in which authors have found a higher prevalence of alcohol drinking among adolescents who participate in sports than among their non-athletic peers [9,47,48,49]. This phenomenon has also been explained by specific sociocultural environments in sports in which young athletes are often in a situation to (1) start consuming alcohol earlier and (2) consume a larger quantity of liquors than non-athletes [47,48]. However, not all sports are uniform in their culture with regard to alcohol consumption. One such sport is almost certainly swimming because of the specific physiological influence of alcohol on the human body. 

Alcohol is a diuretic, and its consumption can result in dehydration. Proper hydration is critical to preventing injuries, creating an optimal environment for building muscle, losing body fat, maximizing energy levels, transporting and absorbing nutrients, and ridding the body of toxins and byproducts [50]. In swimming, proper hydration is additionally important for the following reasons: (1) the gravity effect (i.e., as a result of the lower gravity in the water, blood moves to the chest area, and the kidneys produce more urine to eliminate “excess” water); (2) the temperature effect (because of the lower temperature of the water, blood moves to stomach and inner organs, which is also recognized as an excess of water, resulting in a kidney reaction similar to that previously described); and (3) the pressure effect (because of the higher pressure of the water, the plasma of the blood changes and reacts by being more dense, and thus urine production is stimulated) [51]. Given these deteriorating effects of alcohol drinking on swimming performance, it is reasonable to expect that the consumption of alcohol impairs swimming performance, which means that swimmers who consume alcohol to a greater extent are less successful. Therefore, it is logical to conclude that alcohol consumption is potentially one of the potential causes of poor competitive achievement, which consequently results in even higher susceptibility to doping. 

Although previous discussion where we shortly explained negative influence of alcohol on competitive achievement, and consequent vulnerability to doping in those swimmers who consume alcohol is logical, we can’t ignore the possibility of opposite direction between variables. In short, it is also possible that those swimmers who lack competitive achievement (i.e., sport-result) are at the same time oriented towards (1) alcohol and (2) doping. Indeed, it is possible that for some of the studied swimmers the low competitive achievement is actually “the cause” of both “problematic behaviors” (alcohol consumption and positive doping intentions). By all means, further prospective analyses are needed to clearly identify the cause-effect relationships which exist between these variables. 

### 4.3. Dietary Supplementation and Doping

Dietary supplementation is frequently studied in the context of doping, including the problem of the correlation between knowledge of doping and dietary supplementation [2], as well as the association that may exist between dietary supplementation practices and attitudes toward doping [32]. In general, studies more often did than did not confirm a correlation between the usage of dietary supplements and doping susceptibility in athletes [20,23]. Therefore, although not all studies confirmed the higher doping likelihood in athletes who consume dietary supplements [13], there is widespread opinion that dietary supplementation should be observed as a gateway to doping [32]. Our results, which indicate a higher consumption of dietary supplements in swimmers who are more prone to PDB, confirm the conclusions from previous studies. However, this association must not be simplified. 

Dietary supplementation in sports is very prevalent, and in most cases, it is a natural consequence of the high metabolic demands of athletic training and competition [52,53,54]. In some circumstances, it is strongly suggested even by medical staff (i.e., iron supplementation in female athletes, vitamin-mineral supplementation in youth athletes, and/or protein supplementation in vegans) [54,55,56,57]. Therefore, our results on the higher likelihood of PDB in those who use dietary supplements do not necessary mean that the usage of dietary supplements will later result in doping behavior. However, it is very probable that the correlation between dietary supplementation and doping susceptibility again points to the previously described cause–effect relationship between “dissatisfaction” and “doping susceptibility”. 

Namely, athletes who are not content with their athletic achievement tend to try different methods of overcoming their limitations, including dietary supplementation [8]. Logically, even those who do not achieve their athletic goals after including dietary supplementation in their training regime will, in some circumstances, consider other options, including doping. As a result, it is unlikely that someone will use doping without trying to improve their performance using dietary supplementation first. Therefore, studies repeatedly evidenced a higher likelihood of doping among those who consumed dietary supplements than among those who did not [8,32]. However, it does not allow for an overall generalization of findings, and there is (frequent) stigmatization of dietary supplement users as potentially or currently involved in doping. Namely, while it is very probable that all who are involved in doping are also consumers of dietary supplements, it does not mean that all consumers of dietary supplements are simultaneously potentially and/or currently involved in doping. 

### 4.4. Factors of Hesitation and Doping

With regard to the study aims, one the most important findings pertain to the established associations between the studied factors of hesitation and vulnerability to doping. Of 19 studied variables of hesitation, the significant correlations with PDB were evidenced only for those evidencing the negative social consequences of doping behavior. In brief, swimmers who self-reported negative social consequences as the most important consequences of doping behavior were less likely to be positively and neutrally oriented toward doping behavior. 

To the best of our knowledge, no study has examined the factors of hesitation toward doping behavior in swimming. However, our results are comparable to those of a study that examined factors of hesitation toward doping behavior in college-level athletes involved in different sports [26]. Although differences in statistical analyses do not allow for the absolute comparison of the findings (i.e., for this study, we used multinomial regression analysis, and study conducted among college-level athletes calculated simple correlations), the overall results are similar. In brief, in the study conducted among college-level athletes, the authors evidenced significant correlations between PDB and the variables “familial condemnation” (Spearman’s R = 0.36) and “friends’ condemnation” (R = 0.36), but “self-disappointment” and “condemnation by religious organizations” (church) were also significant correlates of PDB (R: 0.31 and 0.37, respectively). Since our results also indicate strong associations between “negative social consequences” and the likelihood of doping, it seems reasonable to conclude that negative social consequences should be prioritized in anti-doping campaigns. 

Although males were more prone to positive doping behavior, gender was not shown to be a covariate that influences the association between factors of hesitation and doping susceptibility (i.e., all associations found to be significant in “crude” mode, were significant in regression model controlled for gender as cofounding factor). The main explanation for such a finding can be found in characteristic social-environment of swimming sport. In short, male and female swimmers practice together more often than not, while their competitions are organized at the same time (i.e., the disciplines are separated but are organized interchangeably on the same days). This structure logically contributes to similar doping intentions and beliefs, resulting in similar predictors of PDB, as previously discussed, but also in non-significant influence of gender for all of the studied doping-related predictors. 

The non-significant relationships between negative health-related consequences and PDB deserve additional attention. The medical authorities involved in anti-doping campaigns regularly criticize the most common WADA anti-doping approach, which, in general, includes the prevention of athletes from using drugs by a legalistic approach (i.e., sanctioning of the drug users) [4]. Specifically, the medical community suggests an approach that prioritizes athletes’ health [58]. Not surprisingly, anti-doping prevention campaigns often highlight health-related consequences of doping usage [3,6]. Our results, however, do not support such an approach for at least two reasons. First, more than 60% of tested athletes are of the opinion that doping is mainly a problem of “fair play”, and only one-third observe doping as mainly a “health hazard”. Second, the health-related consequences of doping are not significantly related to PDB. 

While only few years ago the majority of athletes regarded doping as mainly a health-related problem, these figures seem to have changed over last years. More specifically, in studies performed 6–8 years ago, sailing athletes, table tennis players, and even swimmers highlighted doping in sports as mainly a health hazard [2,5,19,42]. However, according to the results presented here and those of some very recent studies [20,41], it seems that perceptions have changed, and athletes tend to judge doping as mostly a fair-play issue. One could argue that swimmers observed herein were highly competitive, which could consequently influence their perception of doping as mainly a “fair-play issue”, and this is certainly one of the reasons for such findings, since previous studies where athletes identified doping as health-related problem regularly sampled highly competitive athletes also [2,5,19,42].Therefore, future studies are needed in less competitive and younger athletes. Of course, the interpretation of these trends is beyond the scope of this research, but the findings are indicative. Namely, together with the fact that the health-related consequences of doping behavior were not related do PDB, the prioritization of the fair-play perspective over the health-hazard perspective clearly points to the need for the development of specific anti-doping approaches in swimming. 

### 4.5. Limitations and Strengths of the Study

The study was cross-sectional, and although in some cases, causality between variables can be logically interpreted (i.e., gender is predictor of doping behavior, and not vice versa), the cause–effect relationships in other cases may be questioned (i.e., association between competitive results and doping intentions). Of course, it is unlikely that “low susceptibility to doping” resulted in better athletic achievement, but it is possible that other variables (i.e., motivation, cognitive status) influenced this association as confounding factors. Further, in this study, the covariates included age and gender, and the selection of covariates was based on previous research and the experience of the authors. However, it is possible that the study lacks some other potentially important covariates of doping behavior. Also, it is almost certain that this study did not observe some potentially important predictors of PDB (e.g., motivation). Therefore, in further investigations other factors related to PDB in swimming should be explored. Next, it is possible that athletes tended towards socially desirable answers (i.e., negative tendency toward doping, reported less frequent binge drinking) throughout testing, but this is always a problem of studies of such kind. Meanwhile, we believe that strict anonymity of the testing, and our experience from previous studies decreased this possibility. Finally, the main problem of this research was identification of factors related to PDB, and not PDB “per se”. Therefore, authors are of the opinion that findings of the study, irrespective of limitations, will contribute to knowledge on a field. 

This is one of the first studies to examine the problem of doping behavior while including practically all competitive athletes involved in one sport from the country. It is also probably the first study that has explored the problem of doping in swimming. Additionally, the study used measurement tools that are frequently used in other sports, which allowed for objective comparisons of the obtained results with those previously reported for other sports. 

## 5. Conclusions 

The results evidenced higher doping susceptibility in male swimmers. Further, irrespective of gender and age, swimmers who consumed alcohol to a greater extent, those who used dietary supplementation, and those who achieved lower competitive results showed a higher tendency toward doping behavior in future. As a result, these groups of athletes should be considered target populations in anti-doping campaigns in the sport of swimming. It seems reasonable to observe “low competitive achievement” as the main generating force of all of these relationships, since both alcohol consumption and dietary supplementation could be connected to low achievement in sports. In future studies, the associations among these variables (i.e., consumption of alcohol, dietary supplementation and sport achievement) should be investigated more specifically. 

The majority of swimmers were convinced in presence of doping in their sport, and this is one of the rare studies where athletes’ opinion about doping presence in their sport was not correlated to their doping susceptibility. Additionally, we found no influence of age on doping susceptibility. Irrespective of such a surprising and, we may say, alarming result (i.e., authors expected lower doping susceptibility in younger swimmers), this finding leads us to conclusion that anti-doping efforts in swimming should be equally oriented toward different age categories. Finally, our study evidenced the negative social consequences of doping behavior as the most important factors of hesitation toward PDB. At the same time, health-related and sports-related negative consequences of doping were not related to doping susceptibility, and most swimmers observe doping as mainly a behavior that is against fair play. As a result, the negative social consequences of doping behavior and the issue of fair play should be highlighted in the development of specific and targeted anti-doping campaigns in this sport. 

## Figures and Tables

**Table 1:** Multinomial regression results for potential doping behavior (PDB) with negative PDB as a reference value—sociodemographic and sport-related-predictors (OR—odds ratio; CI—confidence interval).

Predictors	Neutral PDB		Positive PDB	
	OR	95%CI	OR	95%CI
Age (cont)	0.92	0.81–1.06	1.00	0.87–1.15
Gender				
Male	0.76	0.41–1.43	2.77	1.27–6.04
Female	REF		REF	
Education level (cont)	1.54	0.77–3.08	0.95	0.44–2.05
Age when started with swimming (cont)	0.97	0.89–1.08	0.94	0.85–1.04
Experience in swimming (cont)	0.98	0.85–1.13	0.9	0.78–1.05
Competitive result in Olympic disciplines (cont)	0.57	0.41–0.81	0.59	0.39–0.88
Competitive result in non-Olympic disciplines (cont)	0.79	0.56–1.13	0.54	0.35–0.83

LEGEND: cont—indicates variables considered as continuous for the purpose of regression calculation; REF—reference value.

**Table 2:** Multinomial regression results for potential doping behavior (PDB) with negative PDB as a reference value—consumption of dietary supplements, cigarettes, and alcohol, and doping-related predictors (OR—odds ratio; CI—confidence interval).

Predictors	Neutral-PDB		Positive-PDB	
	OR	95%CI	OR	95%CI
Dietary supplementation				
Yes, regularly	1.59	0.58–4.31	3.62	1.25–10.52
From time to time	0.92	0.47–1.84	1.32	0.53–3.20
No	REF		REF	
Alcohol consumption (cont)	2.18	1.08–4.16	2.14	1.05–4.37
Cigarette smoking (cont)	1.16	0.78–1.73	1.16	0.67–1.99
Doping occurrence in swimming (cont)	1.20	0.75–1.93	0.92	0.56–1.51
Number of doping tests (cont)	0.67	0.33–1.39	0.28	0.05–1.42
The main problem of doping in sports				
It is mainly health-threatening behavior	0.91	0.56–5.11	1.11	0.66–5.54
It is against fair play	REF		REF	
Penalties for doping offenders (cont) *	1.62	1.09–2.40	0.77	0.48–1.22
Knowledge on doping (cont)	1.06	0.86–1.29	0.99	0.79–1.25

LEGEND: cont—indicates variables considered as continuous for the purpose of regression calculation; REF—reference value, * higher score indicates less rigid penalties.

**Table 3:** Multinomial regression results for potential doping behavior (PDB) with negative PDB as a reference value—self-rated factors of hesitation against doping (OR—odds ratio; CI—confidence interval).

Predictors	Neutral-PDB		Positive-PDB	
	OR	95%CI	OR	95%CI
Condemnation by family members	0.83	0.67–1.03	0.69	0.56–0.86
Condemnation by friends	0.91	0.73–1.15	0.65	0.52–0.80
Condemnation by the public (beyond family or friends)	1.04	0.85–1.27	0.76	0.63–0.90
Condemnation by religious institutions	1.05	0.88–1.26	0.83	0.64–1.09
The negative image that will be created in media	0.97	0.80–1.17	0.98	0.79–1.21
Underestimation of “clean” results	1.02	0.81–1.28	1.03	0.81–1.31
Possible negative financial consequences	1.02	0.84–1.25	0.98	0.78–1.18
Eventual imprisonment because of the use of illegal drugs	0.89	0.73–1.08	0.96	0.77–1.21
Disqualification from competition	0.94	0.72–1.22	0.96	0.73–1.28
Disqualification of previously achieved results	0.87	0.72–1.06	1.13	0.85–1.49
Potential problems with future employment	0.92	0.72–1.17	0.94	0.72–1.23
Behavioral disorders	0.83	0.67–1.04	0.88	0.69–1.13
Psychological addiction to doping	0.87	0.72–1.05	0.87	0.71–1.08
Hormonal dysfunctions	1.06	0.86–1.32	0.9	0.71–1.14
Problems with the vital organs	1.09	0.84–1.42	0.94	0.72–1.23
Cardiovascular problems	1.09	0.83–1.45	0.93	0.71–1.22
Body deformities	0.93	0.71–1.21	0.92	0.68–1.26
Weakening of immune function	0.85	0.65–1.11	0.83	0.62–1.11
Self-disappointment, or feelings of self-failure	0.86	0.68–1.08	0.75	0.61–0.93

LEGEND: All variables were considered as continuous for the purpose of regression calculation.

**Table 4:** Multivariate multinomial regression results for potential doping behavior (PDB) with negative PDB as a reference value (OR—odds ratio; CI—confidence interval).

Predictors	Neutral-PDB		Positive-PDB	
	OR	95%CI	OR	95%CI
Competitive result in Olympic disciplines (cont)	0.34	0.18–0.63	0.79	0.40–1.56
Competitive result in non-Olympic disciplines (cont)	1.02	0.74–3.20	0.59	0.27–1.27
Dietary supplementation				
Yes, regularly	2.03	0.65–6.15	5.86	1.67–20.62
From time to time	1.19	0.56–2.52	1.92	0.71–5.22
No	REF	REF	REF	REF
Alcohol consumption (cont)	1.87	1.02–3.77	1.73	0.77–3.85
Penalties for doping offenders (cont) *	1.69	1.08–2.63	0.66	0.39–1.14
Condemnation by family members (cont)	0.76	0.56–1.01	0.86	0.63–1.15
Condemnation by friends (cont)	1.05	0.74–1.49	0.77	0.57–1.13
Condemnation by the public—beyond family or friends (cont)	1.19	0.89–1.57	0.90	0.70–1.19

LEGEND: cont—indicates variables considered as continuous for the purpose of regression calculation; REF—reference value; only variables which were significantly correlated to PDB in univariate models were included in this calculation; *—higher score indicates less rigid penalties.

## Supplementary Materials

The following are available online at http://www.mdpi.com/1660-4601/15/8/1720/s1, Table S1: Descriptive statistics (frequencies—F, percentages—%) for categorical and ordinal variables. Table S2: Descriptive statistics (Means and Standard deviations) for parametric variables.
