# Supplementary material for: Identification of Factors Associated with Potential Doping Behavior in Sports: A Cross-Sectional Analysis in High-Level Competitive Swimmers
Source: Int J Environ Res Public Health. 2018 Aug 10;15(8):1720. doi: 10.3390/ijerph15081720 (PMC6121562; doi:10.3390/ijerph15081720)
Supplement: Supplementary file 1 [file ijerph-15-01720-s001.pdf]

Supplementary table 1. Descriptive statistics (frequencies – F, percentages - %) for categorical and ordinal variables

	F	%
**Gender**		
Male	148	49.2%
Female	153	50.8%
**Competitive result in Olympic disciplines**		
regional level	53	17.6%
national championship	227	75.4%
national championship - medal	13	4.3%
European/World/Olympics - participation	8	2.7%
**Competitive result in non-Olympic disciplines**		
regional level	56	18.6%
national championship	85	28.2%
national championship - medal	150	49.8%
European/World - participation	10	3.3%
**Dietary supplementation**		
Yes, regularly	47	15.6%
From time to time	145	48.2%
No	109	36.2%
**Alcohol consumption**		
I don't drink alcohol	212	70.4%
I drink alcohol but never binge	74	24.6%
binge drinking once a month or so	14	4.7%
binging more than once a month	1	0.3%
**Cigarette smoking**		
I don't smoke	294	97.7%
I smoke from time to time, but not daily	3	1.0%
less than 10 cigarettes per day	3	1.0%
more than 10 cigarettes per day	1	0.3%
**Doping occurrence in swimming**		
I don't think doping is used in swimming	8	2.7%
Don't know/not sure	29	9.6%
Doping occurs, but rarely	167	55.5%
Doping is frequent in swimming	97	32.2%
**Number of doping testing**		
Never tested	282	93.7%
Once or twice	14	4.7%
Three times and more	6	2.0%
**The main problem of doping in sports**		
It is mainly health-threatening behavior	112	37.2%
It is against fair play	188	62.5%
**Penalties for doping offenders**		
**lifelong suspension**	72	23.9%
milder punishment for the first time, then lifelong suspension	121	40.2%
suspension for a couple of seasons	101	33.6%
financial punishment	6	2.0%
no punishment/should be allowed	1	0.3%
**Potential doping behavior**		
I would use doping if it would help me (with no negative health	35	11.6%
Not sure	45	15.0%
Don't intend to use it	217	72.1%

Supplementary table 2. Descriptive statistics (Means and Standard deviations) for parametric variables

	Mean	SD
**Sociodemographic and sport factors**		
Age (years)	16.4	2.40
Age when started with swimming (years)	8.7	3.20
Experience in swimming (years)	7.7	2.60
Knowledge on doping (score)	2.80	1.64
**Factors of hesitation ****		
Condemnation by family members	1.78	1.48
Condemnation by friends	1.69	1.45
Condemnation by the public (beyond family or friends)	1.48	1.76
Condemnation by religious institutions	-1.91	1.65
The negative image that will be created in media	1.61	1.65
Underestimation of "clean" results	1.93	1.45
Possible negative financial consequences	1.23	1.70
Eventual imprisonment because of the use of illegal drugs	1.93	1.52
Disqualification from competition	2.44	1.17
Disqualification of previously achieved results	2.00	1.43
Potential problems with future employment	2.31	1.23
Behavioral disorders	2.05	1.35
Psychological addiction to doping	1.92	1.56
Hormonal dysfunctions	1.83	1.54
Problems with the vital organs	2.11	1.29
Cardiovascular problems	2.16	1.24
Body deformities	2.35	1.13
Weakening of immune function	2.31	1.09
Self-disappointment, or feelings of self-failure	2.34	1.35

LEGEND: * - theoretical range for knowledge on doping was from "0" to "10" (maximal score); ** - factors of hesitation were self-rated on a scale ranging from "-3" (not important at all), to "+3" (very important)
